# Integrating olfactory assessments in consciousness disorders: methodologies and therapeutic potential

**DOI:** 10.3389/fneur.2026.1895892

**Published:** 2026-09-03

**Authors:** Shuxian Li, Yuxiu Song, Xiaolan Zhou

**Affiliations:** Department of Rehabilitation, Shengjing Hospital of China Medical University, Shenyang, China

**Keywords:** consciousness assessment, olfactory assessment, olfactory function, prolonged disorder of consciousness, rehabilitation intervention

## Abstract

**Introduction:**

Current clinical assessments for patients with prolonged disorders of consciousness (pDOC) primarily depend on visual and auditory approaches, which often result in misdiagnosis. The olfactory system possesses a distinct anatomical pathway that projects directly to the cortex without thalamic relay, which may retain responsiveness in pDOC patients. Preliminary evidence suggests a link between smelling and consciousness processes, yet there is a gap in applying these findings clinically.

**Methods:**

This review combines insights from neuroanatomy, behavioral neuroscience, and neuromodulation studies to propose a framework based on olfaction for assessing and encouraging consciousness recovery. Literature searches were conducted across PubMed, and Web of Science, for studies published from January 2010 to January 2026, with earlier key works identified through reference screening.

**Results:**

Sensory and cognitively driven sniff response test in pDOC olfactory processing serves as a sensitive biomarker for distinguishing between vegetative and minimally conscious states (MCS) in a single study, and may predict early recovery. Protocols for bedside olfactory discrimination provide quick screening methods but lack multicenter validation. Olfactory training is frequently combined with other senses to improve effectiveness, precluding isolation of its specific contribution. Neuromodulation approaches show preliminary effects on brain rhythms in healthy volunteers, while olfactory electrical stimulation and neurofeedback remain at proof-of-concept stage. No dedicated olfactory rehabilitation protocol has been evaluated in pDOC cohorts.

**Conclusion:**

Olfactory assessment and stimulation represent a promising but nascent frontier in pDOC care. The sniff response test currently has the strongest empirical foundation, yet all approaches require multicenter validation. Future research should prioritize personalized key odor identification, standardized stimulation protocols, and rigorous clinical trials of neuromodulation techniques. Integrating olfactory measures into multimodal strategies may improve diagnostic accuracy and support consciousness recovery.

## 1 Introduction

Prolonged disorders of consciousness (pDOC) refers to impaired consciousness lasting over 28 days following severe traumatic or non-traumatic brain injury (TBI) (1, 2). It primarily includes vegetative state/unresponsive wakefulness syndrome (VS/UWS), and minimally conscious state (MCS). The former is characterized by the coexistence of spontaneous eye opening and sleep–wake cycles, without evidence of command following or purposeful responses to external stimuli. In contrast, patients in MCS exhibit unequivocal non-reflexive behaviors, indicating partial awareness of themselves and their surroundings (1, 2). The Coma Recovery Scale–Revised (CRS-R) remains the gold-standard behavioral assessment tool for differentiating these conditions and detecting subtle signs of consciousness (2, 3). However, it exhibits notable temporal variability, with initial misdiagnosis rates reaching as high as 36% (4); moreover, the administration process is labor-intensive and time-consuming, each assessment takes approximately 25–35 min (5) and requires repeated evaluation at least 5–6 times to improve accuracy (6). Although the Simplified evaluation of CONsciousness disorders (SECONDs) protocol streamlines clinical evaluation (7), its reliability hinges heavily on assessor expertise and remains susceptible to environmental confounders, thereby increasing the risk of overlooking covert signs of consciousness (8). To improve diagnostic accuracy, emerging behavioral markers, such as olfactory responses, have been proposed, though they are not yet incorporated into clinical guidelines (9, 10). These markers probe olfactory awareness, a domain frequently underassessed in routine practice due to limited clinician familiarity and resource constraints (11, 12).

Unlike visual or auditory information, which must first traverse the thalamus route to the primary sensory cortex, the olfactory system constitutes the sole canonical sensory modality that projects directly to the cortex without an obligatory thalamic relay (13, 14). Nevertheless, this anatomical distinction does not signify functional independence from the thalamus. Accumulating evidence has underscores the thalamus's continued involvement in cortical-cortical information processing (15), particularly in cognitive domains such as attention and executive control (16). Clinical investigations reveal a dissociation in olfactory function following unilateral focal thalamic lesions, with impaired olfactory identification but intact basic olfactory detection (17). These characteristics suggest that olfactory responsiveness may be persist despite thalamic dysfunction, holding implications for pDOC diagnosis and management. A clinical study showed that behaviorally meaningful olfactory processing survives severe traumatic brain injury (18), yet whether this reflects conscious processing or subcortical reflexes remains unresolved (19). Furthermore, translating olfactory responses into diagnostic biomarkers is challenged by substantial inter-individual variability (20), which is amplified in brain-injured patients by fluctuating arousal and medications. Sniff-based readouts show promise in differentiating UWS from MCS (18, 21), yet multi-center replication and rigorous validation against established neuroimaging paradigms (e.g., task-based fMRI, EEG-based mental imagery) remain lacking.

Despite the anatomical and functional rationale, the literature on olfaction in pDOC remains fragmented, and no prior review has positioned olfaction as the conceptual center of inquiry. Existing reviews have either focused on olfactory dysfunction in neurodegenerative diseases (22, 23), omitted olfaction from consciousness assessment frameworks (2, 24), or embedded olfactory stimulation within multimodal protocols, precluding evaluation of its specific contribution (25). To address this gap, we systematically examine three questions: whether olfaction influences conscious processing, whether olfactory reactions can differentiate MCS from UWS, and whether olfactory stimulation aids recovery. This review integrates the neuroanatomical, diagnostic, and therapeutic dimensions of olfaction into a unified, pDOC-specific framework, offering a novel and clinically actionable contribution.

## 2 Methods

This review was based on a systematic literature search of PubMed and Web of Science. Search strategies combined terms for disorders of consciousness (e.g., vegetative state, unresponsive wakefulness syndrome, minimally conscious state) with terms for olfaction (e.g., smell, odor, sniff, olfactory stimulation, olfactory evoked potential) and olfactory neuroanatomy/neuromodulation. Reference lists were screened for additional studies. Studies were included if they addressed olfactory–consciousness neuroanatomical links, olfactory diagnostic assessment, therapeutic effects of olfactory stimulation, or mechanistically relevant translational insights. Animal and human studies were eligible, with emphasis on 2010–2025. Studies restricted to olfactory dysfunction in neurodegeneration or COVID-19 without relevance to consciousness, non-English reports without an English abstract, and non-original articles were excluded. Two reviewers independently screened titles and abstracts, and disagreements were resolved by a third reviewer. Owing to heterogeneity, a meta-analysis was not feasible; findings were synthesized narratively and organized around the three core questions: olfactory mechanisms in consciousness, olfactory assessment for diagnosis, and olfactory stimulation for recovery.

## 3 Results

### 3.1 Does the sense of smell influence conscious processes?

Consciousness is a multifaceted phenomenon that includes both the awareness of particular content (conscious content) and the general level of alertness that maintains this awareness (conscious state). This article will outline these two aspects to clarify the profound involvement of olfaction in the construction process of consciousness.

#### 3.1.1 Olfactory involvement in sleep–wake cycle

Normal sleep–wake cycle is one of the indicators of consciousness recovery. Although several classical models, such as the “flip-flop switch Model” and the Two-Process Model, have been proposed to explain the regulation of the sleep–wake cycle (26, 27), the underlying neurobiological mechanisms remain incompletely understood. Wakefulness is not governed by a single brain region, but is sustained by distributed neuronal populations across areas including the Lateral Hypothalamus (LH), the Basal Forebrain (BF), the Ventral Tegmental Area (VTA), the Locus Coeruleus (LC), the Dorsal Raphe Nucleus (DR), the thalamus, and the Parabrachial Nucleus (PBN). Mediated by neurotransmitters such as glutamate and glutamate and gamma-aminobutyric acid (GABA), these populations sustain cortical activation, behavioral arousal, and motivational states via mechanisms including direct excitation, disinhibition, and neuromodulation, while concurrently inhibiting sleep-promoting neurons (28, 29). Sleep comprises non-rapid eye movement (NREM) and rapid eye movement (REM) stages. Building on the distributed arousal networks described above, NREM initiation and maintenance are primarily governed by the ventrolateral preoptic nucleus (VLPO) in the anterior hypothalamus, which contains GABAergic and galaninergic neurons that project to and inhibit major arousal centers [e.g., the LC, DR, and tuberomammillary nucleus (TMN)]. Additional regions including the cortex, thalamus, basal ganglia, medulla, and periaqueductal gray also contribute to NREM regulation (27, 30). REM sleep is orchestrated by brainstem populations, particularly within the pons. REM-on neurons [primarily cholinergic neurons in the pedunculopontine tegmental nucleus (PPT) and laterodorsal tegmental nucleus (LDT) and neurons in the sublaterodorsal nucleus (SLD)] are responsible for cortical desynchronization and muscle atonia; REM-off neurons, located in the extended VLPO and monoaminergic brainstem nuclei, suppress REM initiation (27, 30).

Based on these neural substrates, damage to the aforementioned structures or circuits following severe brain injury represents a primary cause of impaired arousal and disrupted sleep architecture in patients with pDOC. For instance, extensive thalamic lesions or disruption of the ascending reticular activating system (ARAS) can precipitate coma (8, 31). Patients with traumatic brain injury often exhibit decreased orexin levels, contributing to wakefulness deficits (32). Moreover, circadian rhythm disturbances (such as abnormal melatonin secretion) and sleep fragmentation commonly observed in neurocritical patients may further impede neurological recovery and metabolic waste clearance (33, 34). Restoring normal sleep–wake cycles thus serve not only as a marker of consciousness recovery but also as a potential target to promote neuroplasticity and improve clinical outcomes.

**Figure 1:**
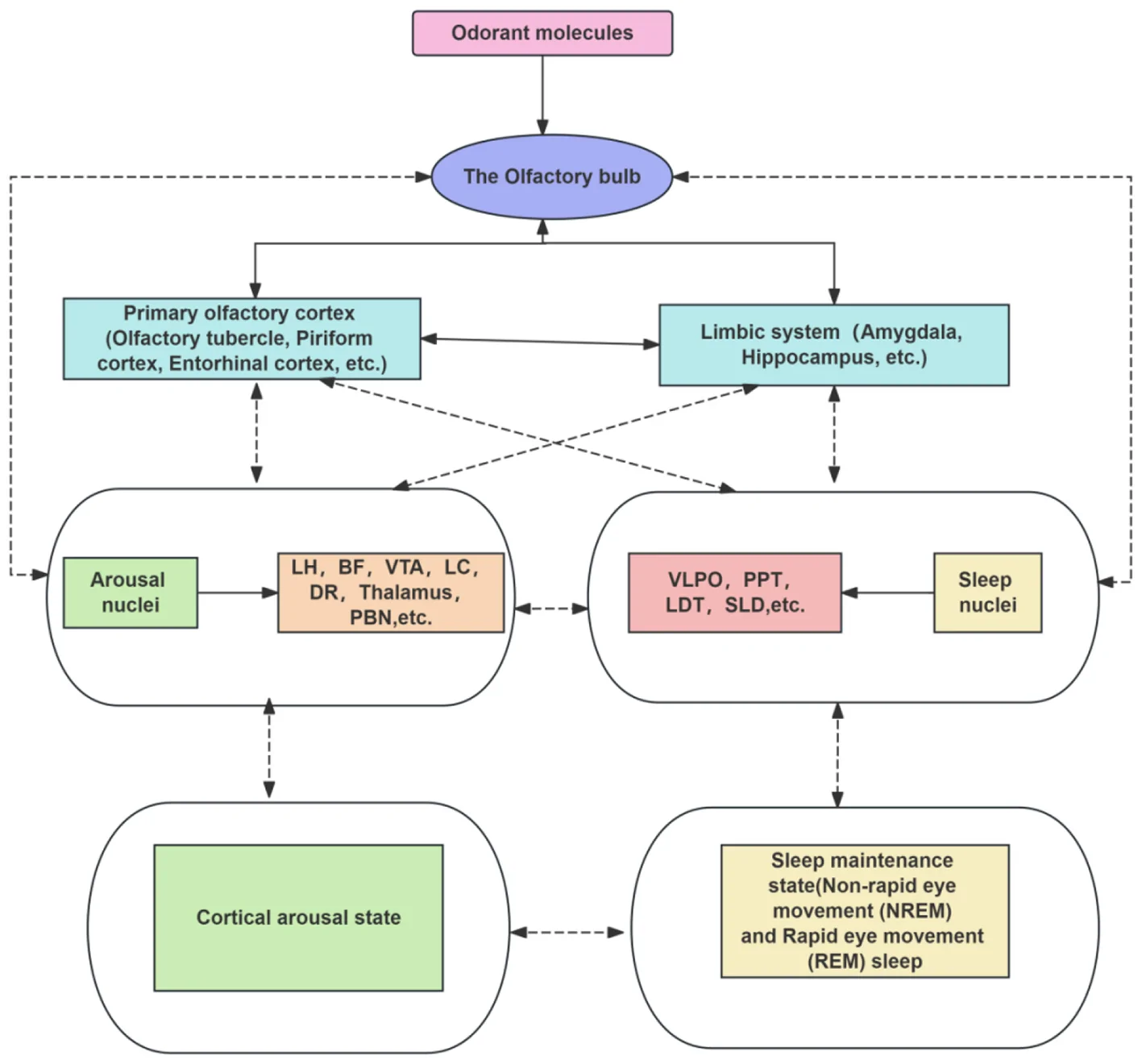
Schematic diagram illustrating the involvement of the olfactory system in the regulation of sleep–wake cycle. Odorant molecules stimulate the olfactory bulb, which then sends signals to the primary olfactory cortex. These areas are also linked to limbic structures. The olfactory pathway then engages with important nuclei that regulate sleep–wake cycles, including areas that promote arousal and those that promote sleep. This interaction among the nuclei helps maintain sleep–wake states. In this schematic, solid lines denote anatomical connections that have been validated across species, including humans. Dashed lines represent functional inferences or indirect modulatory pathways derived primarily from animal studies, which require further validation in humans. LH, lateral hypothalamus; BF, basal forebrain; VTA, ventral tegmental area; LC, locus coeruleus; DR, dorsal raphe nucleus; PBN, parabrachial nucleus; VLPO, ventrolateral preoptic nucleus; PPT, pedunculopontine tegmental nucleus; LDT, laterodorsal tegmental nucleus; SLD, sublaterodorsal nucleus.

The olfactory system establishes extensive and direct connections with multiple subcortical nuclei involved in arousal control, prior to thalamic relay (35–40). For example, the BF cholinergic neurons receive substantial direct inputs from olfactory regions including the olfactory bulb, anterior olfactory nucleus, piriform cortex, entorhinal cortex, basolateral amygdala, and hippocampus; these projections have been characterized primarily in rodents, and their functional significance in the human brain awaits direct confirmation (40, 41). Similarly, the piriform cortex projects directly to the LH and amygdala, and the finding that sleep modulates olfactory memory consolidation via altered piriform cortex activity has been demonstrated in rodents but not yet systematically investigated in humans (42). In contrast, several mechanisms have gained cross-species support. The anteromedial olfactory tubercle highly expresses orexin-1 receptors, enabling the integration of hypothalamic orexin signaling with olfactory input; food-related odors activate dopamine D1 receptor-positive neurons in this region to promote motivated arousal, a mechanism initially identified in mice and consistent with the established role of orexin in human arousal regulation (43). Additionally, the olfactory tubercle receives dense dopaminergic input from the VTA, and activation of this pathway robustly enhances arousal, a circuit demonstrated in rodents (44, 45). Collectively, these findings indicate that the olfactory system participates deeply in sleep–wake modulation through multiple converging mechanisms. Figure 1 presents a schematic diagram of the olfactory system's role in sleep–wake regulation.

It should be noted that the proposed framework in Figure 1 consists of two hierarchical levels. The first level (depicted by solid connections) is based on well-established anatomical projections, such as the olfactory bulb projections to the primary olfactory cortex, piriform cortex, and entorhinal cortex, as well as the direct projections from LH, BF, and LC to cortical arousal systems. The second level (depicted by dashed connections) represents broader theoretical interpretations and functional extrapolations. For instance, how specific odorant signals might modulate sleep–wake switching via limbic or brainstem nuclei. This distinction is intentionally made to clarify that while the anatomical scaffold is conserved across species, the integrative functional roles are currently hypothetical and require direct testing in humans.

Consistent with these mechanistic insights, clinical studies demonstrate that olfactory stimulation exerts bidirectional effects on sleep–wake states. Nocturnal lavender exposure improves sleep efficiency and reduces sleep onset latency in healthy individuals (46) while nighttime rose odor decreases nightmare frequency without disrupting sleep architecture in patients with post-traumatic stress disorder (47). Olfactory signals also modulate memory consolidation through hippocampal theta oscillations; stimulation during sleep enhances theta–spindle coupling, which has been associated with facilitated memory integration, although a direct causal relationship remains to be established (48). Arousal-promoting effects, conversely, depend on the biological relevance of the odorant and the individual's physiological state (49). For instance, citral evokes objective cortical arousal independent of subjective intensity or pleasantness ratings, as reflected by sustained pupillary dilation and EEG spectral shifts (increased beta/gamma, reduced alpha), putatively through odor-induced tonic locus coeruleus (LC) firing and ascending noradrenergic modulation (50). However, the strength of evidence varies considerably. Many studies are small, unblinded, and may be confounded by learned associations and expectancy effects rather than reflecting intrinsic odor properties (20). Yao et al.'s study, while more rigorous, lacks direct causal evidence linking citral to LC activation in humans and is limited to a single odorant in healthy volunteers; alternative mechanisms such as trigeminal co-activation or respiratory modulation cannot be excluded. Moreover, sleep-related findings have largely not been systematically investigated in brain-injured populations, and the proposed therapeutic window of sleep remains underutilized in pDOC (51).

These findings hold translational potential for pDOC but must be interpreted with caution. Carefully selected odorants could serve as adjunctive tools to promote arousal, particularly in patients with post-traumatic hypocretin deficiency (32), while sleep-phase olfactory interventions may offer a non-invasive strategy to enhance sleep-dependent plasticity and memory consolidation (46). Furthermore, citral-induced pupillary and EEG responses, being independent of subjective perception, could provide language-free biomarkers for arousal monitoring in unresponsive patients. Critically, however, evidence supporting all these approaches derives predominantly from healthy volunteers or clinical populations other than pDOC; their feasibility, safety, and efficacy must be rigorously evaluated in dedicated pDOC trials before any clinical application can be recommended.

#### 3.1.2 Olfactory involvement in conscious perceptual integration

##### 3.1.2.1 Levels of olfactory processing

Olfactory responses in brain-injured patients occur at multiple processing levels. These levels form a hierarchy, and clinicians must distinguish them carefully when interpreting the responses' significance for consciousness. At the peripheral level, trigeminal free nerve endings in the nasal mucosa respond to irritant odorants (e.g., ammonia, strong acetic acid). These responses are olfactory-independent and are classified as somatosensory in nature. Furthermore, they do not require cortical integration (52). At the next level, autonomic and reflexive responses, including odor-induced changes in respiratory pattern, heart rate, and skin conductance, are mediated by direct projections from the olfactory bulb to the amygdala and hypothalamus, and can occur without conscious odor perception (53). At the intermediate level, sensory-driven olfactory processing involves the detection, discrimination, and valence evaluation of odors through the primary and secondary olfactory cortices, and may or may not be accompanied by conscious awareness (54, 55). At the highest level, cognitively driven olfactory perception engages prefrontal, hippocampal, and higher-order association networks. This engagement enables conscious odor identification, recognition memory, and context-appropriate behavioral responses (56–59). Determining the processing level of an observed response is critical for interpreting it as evidence of conscious awareness. This is particularly relevant in pDOC patients, where behavioral output is minimal or ambiguous. These four hierarchical levels of olfactory processing are summarized in Table 1.

**Table 1:** Classification of odor-elicited responses by processing level.

Response type	Neural substrate	Conscious awareness required	Clinical significance in pDOC
Trigeminal	Trigeminal nerve endings → spinal trigeminal nucleus → thalamus → somatosensory cortex	No	Indicates intact nasal mucosa and brainstem function; not evidence of olfactory or conscious processing
Autonomic/reflexive	Olfactory bulb → amygdala/hypothalamus → brainstem autonomic nuclei	No	Indicates intact peripheral and subcortical olfactory pathways; may occur without consciousness
Sensory-driven olfactory	Olfactory bulb → piriform cortex → OFC; amygdala, hippocampus	Variable (may or may not be conscious)	Indicates cortical olfactory processing; level 1 (detection) and level 2 (discrimination) differentially preserved in MCS vs. UWS
Cognitively driven olfactory	Prefrontal cortex → hippocampus → limbic system; requires top-down modulation	Yes	Strongest behavioral evidence of conscious awareness; predicts transition from UWS to MCS

OB, olfactory bulb; OFC, orbitofrontal cortex; PFC, prefrontal cortex; MCS, minimally conscious state; UWS, unresponsive wakefulness syndrome.

##### 3.1.2.2 Neuroanatomical basis of olfactory-consciousness network connectivity

The primary olfactory cortex sends projections to the secondary olfactory cortex, forming an indirect thalamocortical route through the dorsomedial thalamic nucleus (16). The orbitofrontal cortex (OFC), as a key secondary olfactory region, receives convergent input from the piriform cortex and the dorsomedial thalamic nucleus, and is involved in odor valuation, multisensory integration, and reward-related processing (60, 61). The secondary olfactory cortex shows strong connections with extensive cortical networks, such as the Default Mode Network (DMN) and the Salience Network (SN) (62, 63). In contrast to the visual and auditory cortices, which need five to six functional connectivity steps to access the main DMN hubs (64), the olfactory cortex integrates similarly within just three to four steps, highlighting its efficient connection with networks related to consciousness (65).

Functional neuroimaging shows that olfactory inputs quickly activate DMN subsystems related to memory, especially the medial prefrontal cortex (mPFC), which gets input from the OFC and anterior cingulate cortex (ACC) and keeps functional links with the hippocampus and posterior cingulate cortex (PCC), aiding in self-referential processing and the recall of odor-related episodes (66, 67). Core SN nodes, such as the ACC and anterior insula, have bidirectional connections with the piriform cortex and OFC, facilitating affective responses to odors and directing attention to olfactory stimuli (61, 68).

The high efficiency of olfactory-DMN connectivity supports the hypothesis that olfactory pathways may serve as particularly resilient conduits for engaging residual conscious processing in the severely injured brain. If validated, this hypothesis would provide a strong rationale for prioritizing olfactory-based paradigms in both diagnostic and therapeutic protocols for pDOC. However, whether this anatomical efficiency translates into functional preservation in pDOC remains to be empirically established.

##### 3.1.2.3 Bilateral synergy and neural plasticity of the olfactory system

The olfactory system demonstrates inherent plasticity and bilateral cooperation, which might support its functional robustness. In animal studies, blocking one nostril leads to structural changes in the olfactory bulb, such as shifts in axon initial segment location and changes in the myelin thickness of the lateral olfactory tract. Significantly, these alterations are observed not only on the sensory-deprived side but also in the contralateral, intact bulb, highlighting system-level plasticity that surpasses unilateral routes (69). Evidence from clinical studies suggests that unilateral olfactory stimulation triggers symmetrical activation in both primary and secondary olfactory cortices, even after eliminating trigeminal and cognitive confounding factors (70). Therefore, although the olfactory system primarily projects ipsilaterally, it also processes information bilaterally in a synergistic manner, providing a theoretical basis for post-injury compensation and recovery. Clinically, this necessitates testing each nostril separately during bedside assessment to avoid false-negative conclusions about residual olfactory capacity. However, the extent to which this bilateral resilience is preserved following the severe diffuse injury characteristic of pDOC remains an open question.

##### 3.1.2.4 Olfactory contributions to multisensory integration

In addition to its inherent ability for independent smell perception, olfaction frequently combines with taste, sight, hearing, and touch to create complex multisensory experiences, which are typical in everyday situations (71, 72). This integration depends on the dynamic functional coordination between different brain areas. For example, in a task where odors are identified using auditory cues, there is increased neural phase synchronization between the auditory cortex and piriform cortex. This synchronization strength is linked to how accurately the task is performed, indicating a potential mechanism for olfactory-auditory integration (73). In a different study that merged psychophysics with fMRI, pure olfactory stimulation, such as phenylethyl alcohol, notably improved the spatial localization of weak tactile stimuli on the same side, along with heightened activity and functional connectivity within the primary olfactory cortex and multisensory association areas, including the OFC, superior temporal sulcus, and inferior parietal lobule (74). These findings mechanistically support the inclusion of olfactory stimulation in multimodal intervention protocols for pDOC, given that cross-modal facilitation may enhance therapeutic effects. However, isolating the specific contribution of olfaction from the aggregate multisensory effect remains a significant methodological challenge.

##### 3.1.2.5 Respiratory coupling: a temporal framework for olfactory-consciousness interaction

Historically, it was thought that the sense of smell only functioned during inhalation and was not continuous (75). Nonetheless, theoretical progress has proposed that it is inherently aligned with the breathing cycle. In mammals, the olfactory bulbs on the left and right sides have symmetrical lateral and medial glomerular maps, each serving unique functions: the lateral map identifies orthonasal signals from the outside environment when inhaling, while the medial map handles retronasal signals that come from within, such as food odors in the mouth during exhalation. These concurrent pathways transmit data to advanced regions to aid in understanding the external world, like recognizing objects, and the internal world, such as monitoring physiological states (76, 77). Significantly, the detection, perception, and decision-making related to smell are closely connected to breathing rhythms, potentially aiding in the temporal alignment of various sensory systems such as vision and hearing, thus supporting the integration of multisensory information. Furthermore, in the latter part of exhalation, advanced brain regions produce top-down cognitive scene signals that simultaneously replay neuronal activity in the olfactory, visual, auditory, and somatosensory cortices, a crucial process for creating emotional scene memories that link the recognition of external objects with internal emotional experiences. Therefore, aside from being a sensory channel, the dual-pathway structure of olfaction (along with coordination of the respiratory phase) offers a mechanistic framework for shifting consciousness between external and internal focus and for dynamic transitions.

**Figure 2:**
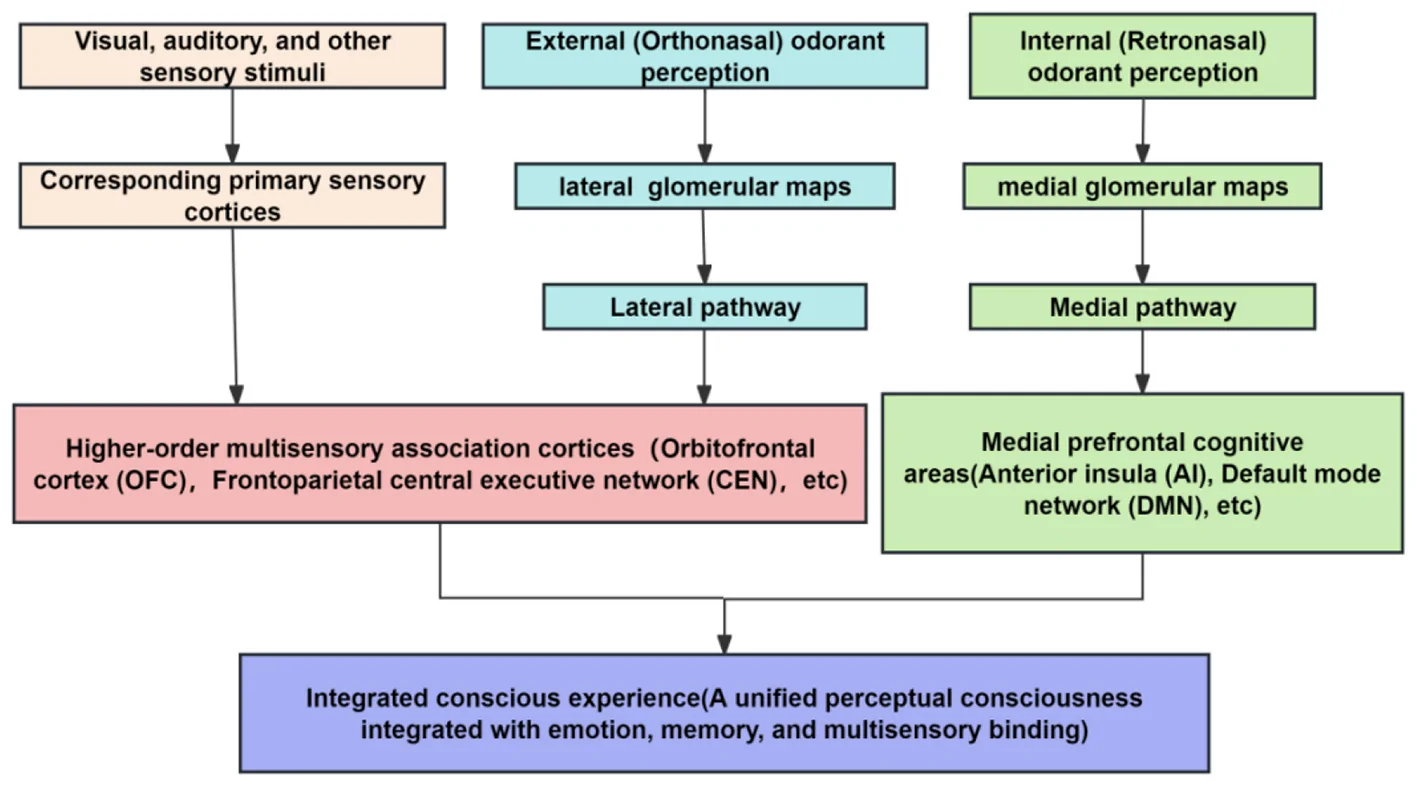
Schematic illustration of olfactory involvement in multisensory integration. External (orthonasal) and internal (retronasal) odorant perception, along with visual, auditory, and other sensory stimuli, activate their respective primary sensory cortices. Olfactory information is processed via lateral and medial glomerular maps, giving rise to lateral and medial pathways. These pathways project to higher-order multisensory association cortices. The convergence of these pathways ultimately contributes to an integrated conscious experience. This framework is adapted from the perspective articles (76, 77). As clearly stated in the original publications, this model remains a working hypothesis rather than an established mechanism, and is presented here to provide a heuristic framework for interpreting the existing anatomical and physiological data and for generating testable predictions.

While this model, schematized in Figure 2, offers an elegant account of respiratory coupled multisensory integration, it must be emphasized that the dual-pathway architecture remains a working hypothesis derived from theoretical proposals, rather than an established mechanism, and direct empirical evidence in humans is consequently still limited. If validated, this dual-pathway model suggests that respiratory phase–locked olfactory stimulation, delivering specific odors selectively during inhalation vs. exhalation, could serve as a targeted intervention to differentially engage exteroceptive and interoceptive conscious processing in patients with pDOC. Concurrently, analysis of spontaneous sniffing patterns may index a patient's attentional orientation toward external stimuli; indeed, sniffing behavior has already been established as a reliable behavioral marker of consciousness in this population (18).

To sum up, the olfactory system is a crucial physiological mechanism that influences both the basic state of consciousness and the details of conscious experience. Despite this, we still lack a comprehensive understanding of how unique olfactory signals influence the transition between sleep and wakefulness, and the exact neural mechanisms involved in multisensory integration with olfaction, which require further validation and detailed study in future research.

### 3.2 Can olfactory reactions help diagnose and differentiate between levels of consciousness?

Evaluating the olfactory reactions in patients with pDOC is difficult because they often cannot communicate or move voluntarily. However, since the olfactory system is the only sensory pathway that directly reaches the cerebral cortex without thalamic relay, this unique neuroanatomical feature confers potential advantages for pDOC patients. Even with extensive damage to the thalamus and in a hypoparoxysmal arousal state, patients' olfactory pathways might remain functional, preserving their ability to respond to odors either behaviorally or electrophysiologically. Moreover, this system offers diagnostic insights that cannot be obtained through other sensory methods. Therefore, this article systematically elaborates on the application of olfactory assessment in patients with pDOC.

#### 3.2.1 Factors confounding olfactory assessment in pDOC

The interpretation of olfactory assessment results in pDOC patients must account for a range of confounding factors that are prevalent in this population and can independently impair olfactory function.

##### 3.2.1.1 Structural olfactory pathway injury

Traumatic brain injury frequently involves damage to olfactory structures, including the olfactory bulb, olfactory tract, cribriform plate, and orbitofrontal cortex. The reported prevalence of post-traumatic olfactory dysfunction ranges from 12% to 30% in moderate-to-severe TBI cohorts (78, 79). In such patients, absent olfactory responses may reflect peripheral damage rather than impaired consciousness, leading to false-negative conclusions about residual awareness.

##### 3.2.1.2 Mechanical ventilation and tracheostomy

Many pDOC patients require prolonged mechanical ventilation or have a tracheostomy, both of which alter nasal airflow patterns and reduce odorant delivery to the olfactory epithelium (80, 81). Furthermore, the presence of a nasogastric tube may physically obstruct one nasal passage, compromising unilateral testing.

##### 3.2.1.3 Sedation and centrally acting medications

Sedatives, antiepileptic drugs, and other neurotropic agents commonly administered in neurocritical care can suppress olfactory processing at multiple levels. Benzodiazepines, for instance, enhance GABAergic inhibition throughout the olfactory pathway (82), while opioids modulate odor perception via receptors expressed in the olfactory bulb and piriform cortex (83). The residual effects of these medications during assessment may dampen or abolish olfactory responses independent of the patient's level of consciousness.

##### 3.2.1.4 Nasal and paranasal disorders

Pre-existing or acquired conditions such as chronic rhinosinusitis, nasal polyposis, mucosal edema from prolonged recumbency, and crusting secondary to inadequate nasal hygiene can obstruct odorant access to the olfactory cleft, producing conductive olfactory loss that confounds central assessment (84).

##### 3.2.1.5 Circadian and arousal fluctuations

Olfactory sensitivity exhibits circadian variation and is modulated by sleep deprivation and arousal level (85). In pDOC patients with severely disrupted sleep–wake cycles (as discussed in Section 2.1), the timing of assessment relative to circadian phase may significantly influence results.

Patient age and etiology also warrant consideration. Olfactory sensitivity declines with age even in healthy individuals (86), which may compound interpretation in older pDOC patients. Furthermore, traumatic brain injury has been associated with more pronounced olfactory cortical activation than anoxic brain injury (ABI) (54), suggesting that the diagnostic sensitivity of olfactory assessment may vary by injury subtype.

Collectively, these factors underscore the necessity of a thorough pre-assessment evaluation, including structural neuroimaging of olfactory pathways, medication review, and otorhinolaryngological examination where feasible, before attributing absent olfactory responses to impaired consciousness. Failure to control for these confounds may substantially undermine the diagnostic specificity of olfactory assessment in pDOC.

#### 3.2.2 Subjective assessment

Presently, clinical tools used to evaluate disorders of consciousness are significantly lacking in olfactory assessment capabilities. The CRS-R does not include a specific subscale for olfactory discrimination (3). Scales such as the WNSSP, SMART, and DOCS include olfactory elements, but their assessment approaches are relatively simple and heavily based on subjective opinions (87–89). The DOCS scale, for instance, utilizes two recognizable odors and categorizes reactions into three types: no response, generalized response (GR), or localized response (LR). An LR is characterized as a reaction to a specific stimulus that happens within 10–15 s, such as oral movements, tongue clicking, or swallowing, a GR involves reflexive or non-specific reactions, and identifying either suggests an olfactory response. This module does not include contrasting stimuli with different valences, such as pleasant vs. unpleasant, nor does it have specific control stimuli like trigeminal stimulation, which restricts its capacity to evaluate odor discrimination beyond basic responsiveness. Moreover, distinguishing between GR and LR relies on how observers interpret the situation. Non-responsiveness might occur due to factors like motor impairment or lack of pain sensitivity, causing false negatives, while environmental stimuli might trigger GR, resulting in false positives (88).

To overcome these challenges, a bedside olfactory discrimination protocol (ODP) was developed, employing four standardized odors (one pleasant, two unpleasant, and ammonia as a trigeminal control) tested bilaterally (90). A positive score requires behavioral reactions (e.g., eye closure, head avoidance) to the trigeminal stimulus and at least one unpleasant odor, with no response to the pleasant odor. The protocol is practical (~5 min, no specialized equipment) and has demonstrated high concordance with fMRI. However, like the DOCS module, ODP relies on observed behavioral responses, introducing interpreter bias, and does not correlate with consciousness level. A further interpretive challenge concerns the use of ammonia: behavioral reactions to trigeminal stimulants can mimic olfactory responses, and many ostensibly olfactory odorants co-activate the trigeminal system (91, 92). In non-communicative patients, distinguishing trigeminal from olfactory processing on behavioral grounds alone is inherently difficult; future protocols should therefore employ stimuli with minimal trigeminal activity.

It must be emphasized that current evidence supporting ODP remains preliminary. The initial validation was single-center with a modest sample size, and fMRI concordance has not been independently replicated. Moreover, the protocol has not been systematically compared with established scales such as the CRS-R, nor evaluated against long-term clinical outcomes. Therefore, ODP should currently be regarded as a promising screening adjunct rather than a standalone diagnostic tool, and its clinical utility awaits confirmation in larger, multi-center studies.

#### 3.2.3 Objective assessment

##### 3.2.3.1 Objective bedside physiological behavioral assessment

Due to the limitations of behavioral olfactory tests, studies have investigated sniff responses as objective physiological indicators for assessing the level of consciousness impairment (18). Sniff responses are measured using nasal cannulae linked to spirometers that track nasal inspiratory volume without requiring patient cooperation (93–95). As outlined in Section 3.1.2, the sniff response paradigm categorizes responses into sensory-driven and cognitively driven types, corresponding to the intermediate and highest levels of the olfactory processing hierarchy, respectively.

Sensory-driven responses involve automatic respiratory changes initiated by odor molecules through direct olfactory pathway activation. Their complexity can be divided into two tiers. Level 1 (odor detection) involves the fundamental recognition of an odor's presence, typically indicated by a noticeable decrease in inhalation volume upon odor exposure. Patients in MCS show significantly reduced initial inspiratory volumes for both pleasant and unpleasant odors relative to baseline, a pattern not observed in UWS patients (18). Level 2 (odor discrimination) is a more advanced response involving valence distinction, where the decrease in inspiratory volume is negatively associated with odor unpleasantness (95, 96). However, only approximately 12.9% of MCS patients exhibit Level 2 responses (18), indicating that processing odor valence necessitates fairly intact cortical–thalamic networks (75).

Cognitively driven responses, in contrast, are initiated by higher-order cognitive functions such as situational comprehension, learning, or anticipation—rather than by actual odor presence. For instance, when patients are shown an empty jar after being told that an odor will be introduced, MCS patients exhibit significantly lower initial inspiratory volumes relative to baseline, whereas UWS patients do not (18). This response is temporally specific (occurring only during the first breath post-stimulus) and requires coordinated activation of the prefrontal cortex, hippocampus, and limbic system. Notably, some UWS patients displaying such responses later transition to MCS, and these sniff responses occasionally emerge before overt behavioral signs, underscoring their potential as early prognostic indicators of consciousness recovery.

The sniff test is simple, non-invasive, and provides objective measurements without active participation, yet its diagnostic accuracy warrants caution. Arzi et al. acknowledged that a substantial proportion of MCS patients fail to show differential sniff responses, meaning that absence of response cannot be equated with unconsciousness. Additionally, structural damage to olfactory pathways (e.g., ethmoid plate, frontal lobe, or orbitofrontal cortex lesions) may yield false negatives. Currently, this test has not been incorporated into any formal clinical guidelines, and its incremental diagnostic value beyond repeated CRS-R assessments remains to be quantified. Therefore, the sniff response should be regarded as a complementary behavioral paradigm with strong neurobiological rationale, positioned within multimodal assessment protocols rather than serving as a standalone diagnostic instrument.

##### 3.2.3.2 Neurophysiological/imaging assessments

Objective neural responses to olfactory stimuli in patients with pDOC can be assessed via complementary neurophysiological [EEG, olfactory event-related potentials (OERPs)] and neuroimaging (fMRI, PET) techniques. Despite limited research on olfactory-evoked EEG in this population, preliminary evidence suggests significant potential (97). A case study utilizing an olfactory imagery approach showed that a patient in MCS could purposefully perform tasks, with performance enhancing through practice (98). In a subsequent prospective cohort of 28 patients with consciousness impairment, individuals were classified as olfactory-responsive (ORES) or non-responsive (N-ORES) based on behavioral responses. EEG metrics—specifically theta connectivity and alpha/beta power—effectively distinguished the groups: ORES patients exhibited EEG patterns resembling healthy controls, whereas N-ORES patients showed increased theta synchronization and reduced alpha/beta activity, indicating more severe network dysfunction and correlating with poor 3-month recovery rates (56, 57). Additionally, nasally-placed electrodes that directly record olfactory bulb EEG activity enable objective assessment of peripheral olfactory function without behavioral feedback; odor-induced theta oscillations comparable to those in healthy individuals provide conclusive evidence of preserved primary olfactory processing (99, 100).

OERP utilizes EEG to measure neural electrical activity in response to olfactory stimuli, concentrating on the latency and amplitude of components like N1, P2, and P3 to evaluate the pathway integrity from peripheral olfactory receptors to the olfactory cortex (101). In a study of 26 patients with post-traumatic anosmia, 17 exhibited detectable OERPs with prolonged N1/P2 latencies and reduced amplitudes—corresponding to milder injuries typically confined to the olfactory bulb on imaging—while the remaining 9 patients without OERPs showed more extensive central damage involving the frontal lobe or orbital gyrus (102). Furthermore, OERP components correlate with spatial and long-term memory functions, suggesting shared neural substrates between olfactory dysfunction and cognitive decline (103). However, no dedicated study has yet examined OERPs specifically in pDOC cohorts.

fMRI evaluates olfactory pathway activation by detecting BOLD signal changes in the olfactory cortex during stimulation. A cross-sectional fMRI study found that primary olfactory processing is substantially preserved in pDOC, with activation levels positively correlating with consciousness severity; etiology modulated these patterns, with traumatic brain injury (TBI) showing the strongest activation and anoxic brain injury (ABI) the weakest (54). ^18^F-FDG PET/CT revealed significantly reduced glucose metabolism in key olfactory regions (bilateral orbitofrontal cortex, thalamus, left hippocampus, and parahippocampal gyrus), reflecting functional disruption, alongside bilaterally increased insular metabolism suggesting compensatory or neuroinflammatory responses. Quantitative analysis identified strong positive correlations between residual functional status and metabolic activity in the right middle frontal orbital gyrus, caudate, and putamen, proposing these regions as potential biomarkers for olfactory preservation and therapeutic intervention (104).

Despite offering complementary objective evidence of olfactory pathway integrity, these techniques remain largely exploratory and have not been validated for individual-level diagnostic classification. Specifically, the EEG study (56, 57) was single-center with 28 patients; OERP has no pDOC-specific data; and the fMRI findings (54), while informative, were cross-sectional without longitudinal follow-up. Sensitivity, specificity, and test-retest reliability in this population remain unestablished. Beyond these method-specific limitations, three cross-cutting challenges impede clinical translation: (i) heterogeneous protocols (odor stimuli, trial numbers, timing) preclude cross-study comparability and meta-analysis; (ii) inter-center reproducibility is largely absent, as most modalities—including EEG/fMRI paradigms and the sniff test awaiting independent replication (18)—remain single-center based; and (iii) olfactory assessment is not yet included in any pDOC clinical guidelines (24). Overcoming these barriers requires large-scale, multicenter prospective studies with pre-registered protocols and blinded outcome adjudication to establish robust diagnostic and prognostic utility. Table 2 provides a summary of reviewed methods and their clinical validation levels.

**Table 2:** Summary of olfactory assessment methods in pDOC patients.

Assessment category	Method	Measurement content	MCS vs. UWS differentiation	Main advantages	Current level of clinical validation	Key limitations
Behavioral assessment	DOCS	Behavioral responses to 2 familiar odors	No	Quick bedside	Single-center validation (88) (*n* = 23); no independent replication	Relies on behavioral observation; interpreter bias; does not correlate with consciousness level
	ODP	Use of 4 standardized odors to calculate ODP index (0/1)	No	Standardized controls; higher specificity than DOCS module	Single-center exploratory study (90) (*n* = 16); no independent replication	
Psychophysiological assessment	Sniff responses test	Changes in nasal inspiratory volume during olfactory stimuli	Yes	Objective, non-invasive; can detect covert consciousness	Single-center exploratory study (18) (*n* = 50); no independent replication	False negative risk; diagnostic accuracy not fully established; not yet incorporated into clinical guidelines
Neurophysiological/neuroimaging assessment	Olfactory EEG and related paradigms	Olfactory-related neural activity (e.g., OERP, olfactory imagery tasks)	Yes	Direct neural evidence, prognostic	Single-center exploratory study (56, 57) (*n* = 28); no independent replication	Not yet validated for individual-level classification; limited pDOC-specific data
	Olfactory fMRI	BOLD signal changes in brain regions during olfactory stimuli	Yes	High spatial resolution, whole-brain mapping	Single-center cross-sectional study (54) (*n* = 33); no independent replication	Expensive; not portable; contraindicated in patients with metal implants; limited temporal resolution
	Olfactory PET	Regional glucose metabolism	Yes	Quantitative metabolic data	Single-center study (104) (*n* = 15); no independent replication	Radiation exposure; high cost; limited availability; not suitable for repeated assessments

DOCS, disorders of consciousness scale; ODP, olfactory discrimination protocol; EEG, electroencephalography; fMRI, functional magnetic resonance imaging; PET, positron emission tomography; OERP, olfactory event-related potentials; BOLD, blood-oxygen-level-dependent; MCS, minimally conscious state; UWS, unresponsive wakefulness syndrome.

### 3.3 Can olfaction stimulation serve as an effective tool for recovery?

#### 3.3.1 Traditional olfactory training techniques

Research conducted in healthy individuals and non-pDOC clinical populations has indicates that olfactory training enhances cognitive abilities (105). Certain studies in healthy adults propose that opting for familiar and emotionally significant odors, particularly those tied to personal experiences, might more effectively evoke nostalgia and lead to positive psychological impacts (106). On the other hand, different protocols that involve exposing individuals to seven unique scents for 2 h each week have been found to improve memory and brain function (107). A comprehensive review suggests that olfactory training is linked to overall cognitive enhancement, as well as increased structural volume and changes in functional connectivity in regions related to smell, like the olfactory bulb and hippocampus. Importantly, these positive effects are not limited to people with olfactory impairment or anosmia but also apply to those with normal olfactory abilities (108). Critically, all evidence derives from populations without disorders of consciousness, and whether olfactory training exerts comparable effects in the severely injured brain remains unknown.

In clinical practice, olfactory stimulation is often embedded within multimodal sensory programs for pDOC patients, with some evidence suggesting potential benefits for consciousness (109, 110). However, these interventions largely rely on empirically chosen familiar scents and lack theoretically grounded, systematically developed protocols; the therapeutic value of olfactory stimulation in pDOC thus remains a plausible but unsubstantiated hypothesis. This uncertainty is compounded by the methodological limitations of the existing evidence, which derives predominantly from small, uncontrolled case series and single-arm pre-post designs, precluding causal attribution of observed improvements to olfactory stimulation specifically (109). Furthermore, the multifactorial nature of multimodal interventions—in which olfactory, auditory, tactile, and proprioceptive stimuli are delivered concurrently—makes it difficult to determine whether any observed benefit reflects olfactory-specific effects or the cumulative impact of enriched sensory environments (110). An additional concern is that highly arousing or trigeminal stimuli (e.g., ammonia) are sometimes employed to elicit maximal behavioral responses, yet these primarily activate the trigeminal system, and the resulting reflexive responses should not be interpreted as olfactory processing or conscious perception. For interventions aiming to engage olfactory-specific circuits, the use of pure olfactory stimuli with minimal trigeminal co-activation is therefore recommended. Future studies should combine objective olfactory assessments with systematic odorant selection to identify scents that reliably evoke neural responses and to develop theoretically informed, standardized stimulation protocols for this population.

#### 3.3.2 Olfactory-related neuromodulation approaches

Recently, there has been extensive research into non-invasive neuromodulation techniques for pDOC patients, with multimodal strategies showing promising results (111). Several olfactory-related neuromodulation approaches have been explored at the theoretical or preliminary experimental level, though none has been specifically tested in pDOC cohorts.

##### 3.3.2.1 Ultra-slow frequency olfactory stimulation

Piarulli et al. (112) demonstrated in healthy volunteers that 0.05 Hz electrical stimulation of the olfactory epithelium modulates brain rhythms by enhancing delta and theta activity. However, this finding is derived from a single experimental study in healthy subjects and has not been replicated or extended to brain-injured populations.

##### 3.3.2.2 Rhythmic nasal airflow stimulation

Salimi et al. (113) reported that 0.2 Hz rhythmic stimulation of nasal airflow increases gamma power within the default mode network, improves network synchrony, and elevates EEG complexity in healthy individuals. These results, while mechanistically interesting, are preliminary and rest on small-sample data without clinical validation.

##### 3.3.2.3 Non-invasive olfactory electrical stimulation

Cakmak et al. (114) proposed a wearable olfactory electrical stimulation protocol (Montage6) suitable for extended use. To date, this protocol has been supported primarily by computational simulations; empirical testing in any clinical population, including pDOC, is absent.

##### 3.3.2.4 Olfactory neurofeedback

Olfactory neurofeedback, a brain-computer interface approach enabling self-regulation via EEG-driven olfactory stimulation, remains at the proof-of-concept stage. Initial reports describe system architecture and feasibility in healthy volunteers (115–117), but no clinical trial data are available.

In conclusion, the therapeutic application of olfactory stimulation in pDOC remains at a nascent stage. For both olfactory training and neuromodulation approaches, the current evidence derives predominantly from healthy populations or preclinical models, and dedicated studies in pDOC cohorts are virtually absent. Future research should prioritize two complementary directions: first, the use of objective olfactory assessments to identify personalized “key odors” capable of reliably evoking neural responses in individual patients; and second, the rigorous validation of stimulation parameters and therapeutic efficacy through well-controlled, prospective clinical trials. Table 3 provides an overview of the olfactory training and neuromodulation methods reviewed, along with their current levels of clinical validation.

**Table 3:** Summary of olfactory-based interventions and neuromodulation approaches for pDOC: evidence level and validation status.

Intervention	Study design	Key features	Main findings	Stage of validation	Principal limitations
Familiar/emotionally significant odor exposure	Experimental; cross-sectional	Nostalgia-evoking odors tied to personal experience	Reported to evoke nostalgia and positive mood (106) (*n* = 160)	Non-pDOC clinical	No pDOC data; effects may depend on intact autobiographical memory
Overnight olfactory enrichment	Single RCT	Seven odors delivered via diffuser; 2 h/night for 4 months	Associated with improved memory and modified uncinate fasciculus integrity (107) (*n* = 43)	Non-pDOC clinical	No pDOC or brain-injury data; single study
Structured olfactory training	Systematic review of heterogeneous studies	Twice-daily exposure to four odors over 12+ weeks	Associated with global cognitive enhancement, increased olfactory bulb volume, and altered functional connectivity (108)	Non-pDOC clinical	Benefits in pDOC entirely unexamined
Multimodal sensory stimulation (including olfactory)	Systematic review of heterogeneous studies; single experimental study	Empirically chosen familiar scents within multisensory protocols	Some studies report improved consciousness levels (110) (*n* = 23); olfactory-specific contribution cannot be isolated (109)	pDOC exploratory	No RCT evidence; uncontrolled designs; low certainty
Ultra-slow frequency (0.05 Hz) olfactory stimulation	Single experimental study	Electrical stimulation of olfactory epithelium	Reported to enhance delta and theta activity (112) (*n* = 20)	Healthy volunteers only	No clinical replication; not tested in any patient population
Rhythmic nasal airflow stimulation (0.2 Hz)	Single experimental study	Rhythmic nasal airflow via nasal cannula	Associated with increased DMN gamma power and improved network synchrony (113) (*n* = 15)	Healthy volunteers only	Small sample; no clinical data
Olfactory electrical stimulation (Montage6)	Computational simulation	Wearable non-invasive olfactory electrical stimulation	Conceptual proposal supported by computational simulations (114)	Preclinical/theoretical	No empirical testing in any population
Olfactory neurofeedback	Proof-of-concept; technical reports	EEG-driven olfactory stimulation for self-regulation	Preliminary feasibility demonstrated in very small samples (115–117)	Healthy volunteers only	Proof-of-concept stage; substantial technical barriers to pDOC application

pDOC, prolonged disorders of consciousness; RCT, randomized controlled trial; DMN, default mode network; EEG, electroencephalography; N/A, not applicable.

All findings derive from the populations indicated. No dedicated olfactory rehabilitation protocol has been systematically evaluated in a pDOC cohort. The applicability of these approaches to pDOC remains hypothetical and requires prospective validation. Stage of validation categories: Preclinical/Theoretical (computational or animal models only), Healthy volunteers only (no clinical population tested), Non-pDOC clinical (tested in clinical populations other than pDOC), pDOC exploratory (preliminary studies in pDOC without independent replication).

## 4 Summary and future directions

This review has examined three interconnected questions regarding the role of olfaction in pDOC: the neurobiological mechanisms linking olfaction to consciousness, the diagnostic utility of olfactory assessment, and the therapeutic potential of olfactory stimulation.

The available evidence indicates that the olfactory system is anatomically and functionally positioned to influence both the level and content of consciousness. Its direct access to subcortical arousal centers and high-efficiency connectivity with higher-order networks such as the DMN and SN provide a plausible mechanistic basis for olfactory-mediated modulation of sleep–wake cycles and conscious perception. Clinically, olfactory assessment, ranging from bedside behavioral protocols such as the ODP, through objective sniff response testing, to neurophysiological and neuroimaging techniques, has demonstrated the capacity to detect residual olfactory function and, in some cases, covert awareness not captured by standard neurobehavioral scales. However, the translation of these findings into routine practice is constrained by the preliminary nature of the evidence, the absence of standardized protocols, and a range of confounding factors prevalent in pDOC populations.

### 4.1 Future research priorities

To advance the field, we propose that future investigations be prioritized according to both expected clinical impact and feasibility:

1. High priority: Independent replication and multicenter validation of the sniff test. Among the assessment methods reviewed, the sniff response test (18) has the strongest empirical foundation, it relies on simple and widely available equipment and yields objective physiological readouts without requiring patient cooperation. However, it has not yet been independently replicated. A large-scale, prospective, multicenter trial with blinded outcome adjudication is the most urgent next step toward establishing its diagnostic accuracy and incremental value beyond repeated CRS-R assessments.
2. High priority: Personalized key odor identification. The identification of odors that reliably evoke neural responses in individual patients, based on objective assessments such as sniff parameters, EEG, or fMRI, would enable the design of personalized stimulation protocols. This approach, which leverages each patient's residual olfactory capacity, is both clinically feasible and mechanistically grounded, and could directly inform the development of targeted olfactory training and neuromodulation interventions. As discussed above, the specificity of this selection process hinges on distinguishing olfactory-specific processing from non-specific trigeminal or autonomic reactions—a critical step for ensuring that the identified stimuli engage olfactory networks rather than broader arousal or somatosensory systems.
3. Medium priority: Development of standardized olfactory assessment and intervention protocols. Consensus-based guidelines for stimulus selection, delivery methods, outcome measurement, and reporting standards are essential for cross-study comparability and eventual clinical implementation. Such standardization efforts should involve multidisciplinary collaboration across neurology, otorhinolaryngology, and neurorehabilitation, and should explicitly address the confounding factors identified in this review, including structural olfactory pathway injury, mechanical ventilation, sedation, and nasal comorbidities.
4. Medium priority: Clinical validation of olfactory neuromodulation techniques. Techniques such as ultra-slow frequency olfactory stimulation, rhythmic nasal airflow stimulation, and olfactory electrical stimulation have demonstrated intriguing effects on brain rhythms and network synchrony in healthy individuals. Their translation to pDOC will require a stepwise approach: first, establishing safety and feasibility in small pDOC cohorts; second, identifying optimal stimulation parameters; and third, evaluating therapeutic efficacy through controlled clinical trials.
5. Lower priority: Olfactory neurofeedback. Olfactory neurofeedback remains at the proof-of-concept stage even in healthy volunteers. Given the substantial technical and logistical barriers to its implementation in pDOC, it represents a longer-term research direction rather than an immediate clinical priority.

In conclusion, olfactory assessment and stimulation represent a promising frontier in the diagnosis and rehabilitation of pDOC, grounded in a unique neurobiological rationale. However, the field must transition from single-center exploratory studies to large-scale, methodologically rigorous, and collaboratively executed research programs. The prioritization framework proposed above is intended to guide this transition, with the ultimate goal of integrating olfactory-based approaches into the standard clinical management pathway for patients with disorders of consciousness.
